# Medial migration of the helical blade with penetration into the acetabulum: a rare complication using the TFNA nail

**DOI:** 10.1007/s00590-023-03615-w

**Published:** 2023-06-23

**Authors:** Adrian Deichsel, J. Christoph Katthagen, Michael J. Raschke, Oliver Riesenbeck

**Affiliations:** Department of Trauma, Hand and Reconstructive Surgery, University Hospital Münster, Albert-Schweitzer-Campus 1, Building W1, 48149 Münster, Germany

**Keywords:** TFNA, Femoral nailing, Complication, Cutout, Medial migration

## Abstract

**Purpose:**

To determine the frequency and possible reasons of medial migration with penetration into the acetabulum (MMPA) of the helical blade when using the Trochanteric Fixation Nail Advanced (TFNA) is used for treatment of pertrochanteric fractures.

**Methods:**

All patients with pertrochanteric femoral fracture, treated by intramedullary femoral nailing with the TFNA, were retrospectively reviewed for MMPA of the helical blade. Epidemiological parameters, additional procedures, distance of medial migration, time from primary operation to revision as well as type of revision were assessed.

**Results:**

4 of 153 patients treated with the TFNA developed an MMPA of the helical blade (risk = 2.6%), with a mean medial migration of the blade of 11.6 mm (SD 8.8). The mean time from initial operation to revision surgery was 70 days (SD 30). All patients were revised by conversion to cemented total hip arthroplasty.

**Conclusion:**

MMPA of the helical blade is a rare but potentially hazardous complication of femoral nailing with the TFNA femoral nail, resulting in the necessity for revision surgery and total hip arthroplasty.

## Introduction

Fractures of the proximal femur, of which petrochanteric fractures are the most frequent, account for a substantial part of all fractures, with a rising incidence, especially in the elderly population [1]. To limit the surgery-related trauma and allow early weight-bearing, these fractures are typically treated with intramedullary femoral nails (IFNs) [2]. Different types of implants by various manufacturers are currently available for clinical use [3]. A key design feature differing the currently available IFNs is the fixation of the femoral head and neck by use of Lag screws, dynamic screws, or blades. The femoral head element (FHE) can slide in the nail, allowing a dynamic compression of the fracture under load [4]. The Trochanteric Fixation Nail Advanced (TFNA; DePuy Synthes, Raynham, USA) is one of the frequently used IFNs for surgical treatment of pertrochanteric femoral fractures [5]. It allows the use of both dynamic screws as well as helical blades for fixation of the femoral head. The helical blade impacts cancellous bone and increases stability in osteoporosis. In addition, augmentation with PMMA cement is a further option for increased primary stability.

Complications after intramedullary nailing of pertrochanteric fractures have been extensively reported in the literature [6]. The most frequently observed postoperative mechanical complication is a cutout of the femoral head element through the superior portion of the femoral head, with varus collapse of the fracture and need for subsequent revision surgery [7, 8]. The use of helical blades was previously shown to possess higher biomechanical resistance against cutout, in comparison with dynamic screws [8, 9]. A sparsely reported complication after intramedullary nailing of pertrochanteric fractures is an atypical protrusion of the blade in which the femoral head element exits the femoral head medially. This phenomenon can occur with medial migration of the femoral head element in the intramedullary nail, contrary to the dynamic mechanism of the nail. Subsequently, medial migration with penetration into the acetabulum (MMPA) can occur. In the literature, few cases of MMPA have been reported, typically in the form of case reports [3, 10–12].

The aim of this study was to assess whether MMPA occurs when using the TFNA, and if, so the frequency of this complication.

## Materials and methods

### Retrospective chart review

This study was reviewed and approved by the ethical board of the University of Münster (IRB No. 2022-393-f-S). The TFNA was introduced at our level 1 trauma center in 2019 and was used exclusively with helical blades for the treatment of pertrochanteric fractures (AO/OTA 31A1–31A2). Patients treated with a TFNA from January 2019 to January 2022 were identified from the digital hospital documentation system. Patients treated with a TFNA were screened for symptomatic MMPA. If MMPA was present, the patient was included into the study. The absolute risk was calculated by dividing the number of patients with MMPA by the total number of patients treated with a TFNA. Age at primary implantation of the TFNA, sex, height, weight, and BMI was recorded. The type of fracture before implantation of the nail was classified according to the AO/OTA classification of fractures [13]. The operation report was screened for additional procedures (e.g., cement augmentation or cerclages of the femoral shaft), performed concomitant to the nail implantation, perioperative complications, as well as signs of perioperative problems with the implant. The time from primary implantation to revision surgery, as well as the type of revision surgery, was assessed. Furthermore, relevant comorbidities were recorded.

### Quantification of tip-apex distance and distance of medial migration in the intramedullary nail

The tip-apex distance (TAD) was determined, according to the method proposed by Baumgaertner et al. [14]. Briefly, the point at which a line drawn through the center of the femoral neck intersected the subchondral bone was marked of the apex femoral head. The distance to the tip of the blade was measured on standing ap and lateral hip X-rays and added to result in the TAD.

**Fig. 1:**
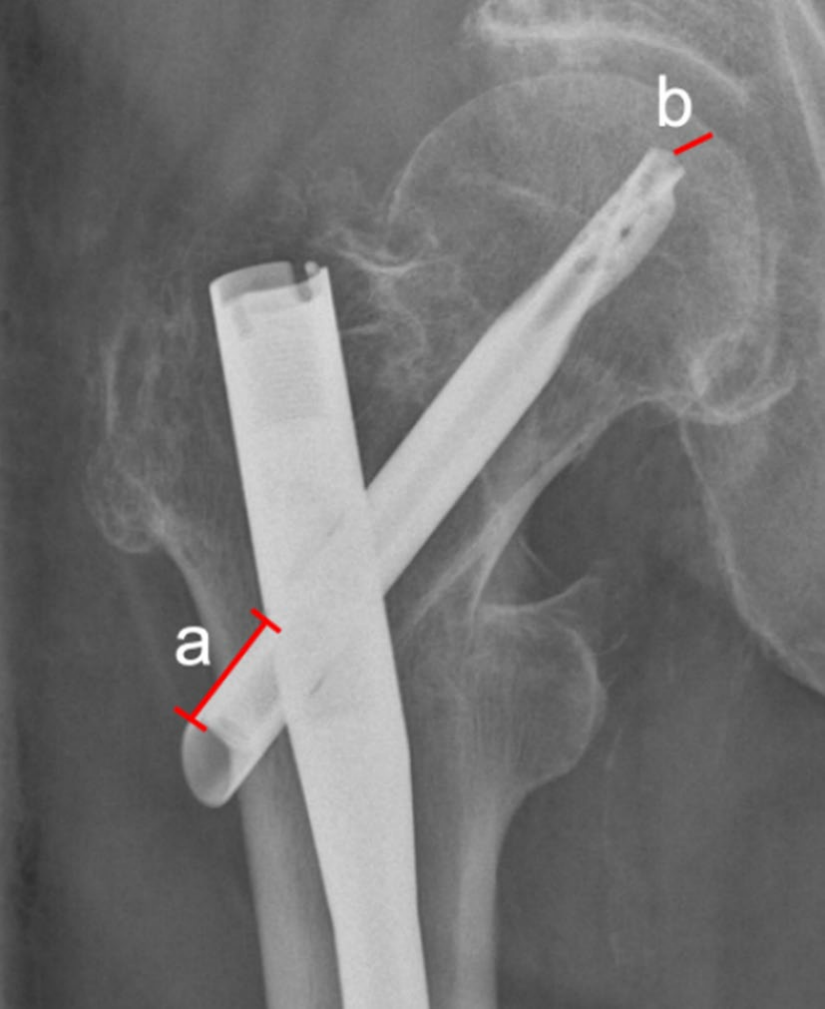
A straight line running from the lateral superior edge of the helical blade to the rim of the femoral nail was drawn and the distance (a) noted. The difference in distance between the postoperative X-rays and the X-rays immediately before revision corresponds the medial migration of the blade in the nail. Tip-apex distance was measured by drawing a line from the tip of the blade to the apex of the femoral head (b)

The migration in medial direction of the helical blade in the hole of the femoral nail was quantified as follows: Anteroposterior (ap) pelvis X-rays, immediately postoperative after primary implantation of the femoral nail, and before revision surgery were used for quantification. A straight line running from the lateral superior edge of the helical blade to the rim of the femoral nail was drawn and the length noted (Fig. 1). The difference in length between the postoperative and pre-revision X-rays corresponds the medial migration of the blade in the nail.

Descriptive statistics of the data was performed using PRISM (version 8, GraphPad Software, San Diego, USA). The results are presented as mean values and standard deviation (SD).

## Results

**Fig. 2:**
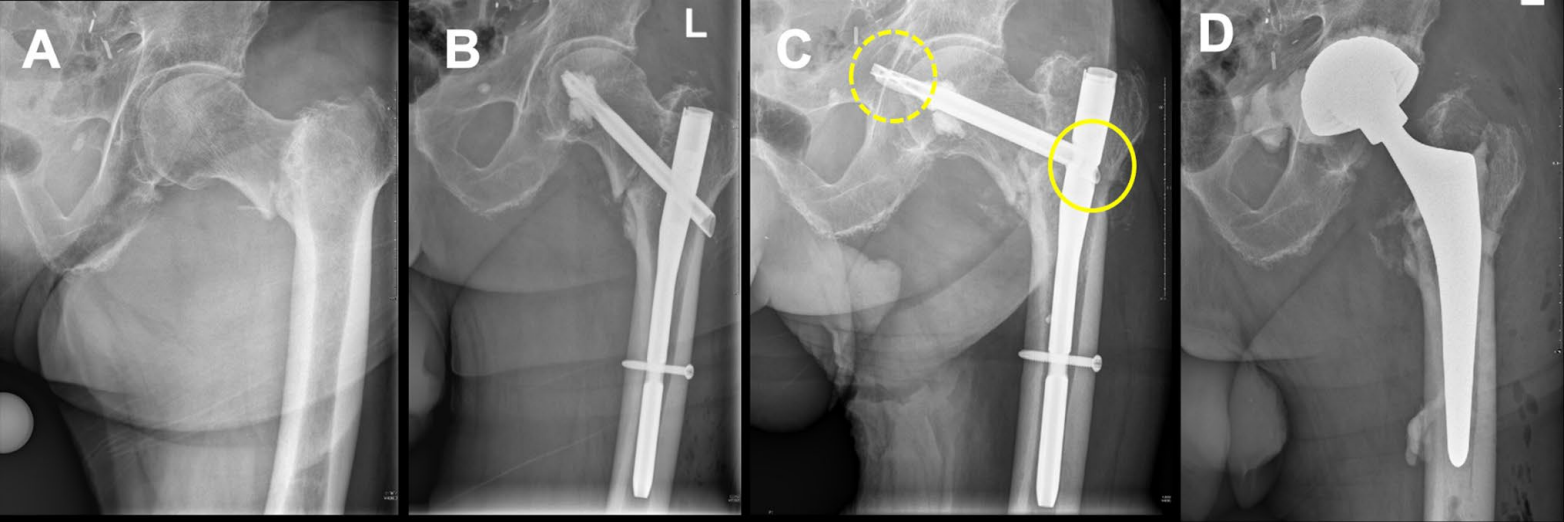
**a** 92 years old, male patient, with pertrochanteric femur fracture. **b** Closed reduction and internal fixation with a Trochanteric Fixation Nail Advanced (TFNA) with helical blade. Cement augmentation was performed, due to poor bone quality. **c** After 72 days, the patient presented with immobilizing hip pain. The helical blade was found to have migrated medially in the intramedullary nail (closed circle) with penetration through the acetabulum and into the small pelvis (dotted circle). **d** Revision surgery was performed with cemented total hip arthroplasty

From January 2019 to January 2022, 153 patients with pertrochanteric fracture were treated with a TFNA at our institution. All patients were treated with a helical blade for fixation of the femoral head and neck. After chart review, 4 patients with MMPA were identified (absolute risk = 2.6%). For all patients, every parameter of interest was available for assessment. The demographics of the patients can be found in Table 1. Age at primary operation was 85 years (SD 8.4). The treated fractures were classified as AO 31A1 in one case and as AO 31A2 in three cases. Overall, the TFNA was used for the treatment of AO 31A1 to AO 31A3 fractures. Primary operations were performed by senior surgeons only. No intraoperative complications, or indicators of faulty implant handling, were described during initial implantation of the nail. In two cases, protective cerclages of the femoral shaft were applied. In one case, cement augmentation due to poor bone quality of the femoral head and neck was performed. The TAD immediately postoperatively, in patients which later presented with MMPA, was 19.2 mm (SD 6.2). The helical blade migrated medial in the femoral nail with a mean of 11.6 mm (SD 8.8). In all four cases, the helical blade penetrated through the acetabulum. However, no vascular or intestinal complications were reported. All patients presented with pain and movement restriction of the hip joint. Revision surgery was performed after a mean of 70 days (SD 30). All patients underwent revision surgery by total hip arthroplasty, with cemented acetabular cup in all cases (Fig. 2). No perioperative complications occurred during revision surgery.

**Table 1:** Demographics of included patients

Patient	Age	Sex	Weight (kg)	Height (cm)	BMI	Fracture type (AO/OTA)	Smoking	Time to revision (d)
1	92	M	68	166	24	31.A1	No	72
2	73	F	78	160	30	31.A2	No	89
3	89	F	75	158	30	31.A2	No	26
4	87	F	75	167	26	31.A2	No	92

*BMI* Body-Mass-Index

## Discussion

The main finding of this study is that MMPA is a rare complication in patients with pertrochanteric fractures treated with the TFNA system and helical blades. To our knowledge, this is the first study to describe MMPA after femoral nailing in patients treated solely with the TFNA system and helical blades.

A cutout as a complication, when using intramedullary femoral nails for treatment of pertrochanteric fractures, was originally defined as a superolateral cutout of a lag screw with varus collapse of the fracture [7]. To prevent cutouts, helical blades were developed, theoretically removing less bone than a screw, as well as compacting the intramedullary bone to improve local bone quality. Initial biomechanical studies confirmed these hypotheses by showing an improved resistance to cutout, in comparison with lag screws [8]. However, clinical studies failed to verify these benefits, showing either comparable, or inferior rates of cutouts when using helical blades, in comparison with screws [12]. Furthermore, a new type of complication characterized by “medial cutout” or “cut through” of the helical blade from the femoral head was shown to become prevalent with the use of helical blades. It was shown that helical blades showed cutout in medial direction significantly more often than lag screws [15]. Although only few reports exist in the literature, different hypotheses as to the genesis of medial migration exist. Talia et al. hypothesized that a failure of the sliding mechanism of the femoral head element might promote medial cutout [12]. A biomechanic study by Weil et al. was performed to determine the reasons for medial migration of the femoral head element [16]. In a laboratory model simulating an unstable fracture with insufficient calcar support, the authors were able to replicate a medial migration of the femoral head element in five different nail types (TFNA not included). It was hypothesized that toggling of the femoral head element in the femoral nail under loading, due to fractures with insufficient lateral cortical, and unstable calcar support, is necessary for medial migration to occur. In the patients with MMPA included in this study, reduced lateral cortical support and an unstable calcar fracture pattern may be hypothesized, based on preoperative radiographs in 1 and 3 cases, respectively, possibly supporting the above-mentioned hypothesis. A possible hardware-based solution for excessive medial migration of the FHE might be the addition of a rim with increased diameter at the lateral side of the FHE, which would prevent the FHE from passing completely through the nail.

This study specifically searched for cases in which a medial migration of the helical blade in the nail occurred, MMPA, which poses the worst-case scenario of medial cutout. Isolated Cases of MMPA have been previously described in the literature for both helical blades as well as lag screws. Reports are published for the Gamma3 [11] (Stryker, Mahwah, USA), the trochanteric nail [17] (DePuy ACE, Raynham, USA), the proximal femur nail [3], and several others. In most of the patients previously described, a screw was used as FHE, while the patient collective in this study was treated by use of a helical blade. However, it can be stated, that the occurrence MMPA is not exclusive to the TFNA but occurs in different implant designs with different types of FHEs.

Another possible factor influencing cutouts is the TAD. It has been generally accepted that a high TAD increases the risk for cutouts. Typically, a TAD of < 20–25 mm is recommended, to decrease the risk for cutouts [14]. Recent studies questioned this general recommendation for the use of systems utilizing helical blades, showing that a TAD < 20 mm might increase the risk for cutouts in the TFN and PFNA system [18]. However, these studies investigated classic cutout, not MMPA. In our patients, mean postoperative TAD was 19.2 mm. Whether this influenced the risk for MMPA remains unclear and may be elucidated in further studies.

Initial studies on the outcome of the TFNA in proximal femoral fractures reported good outcome [5]. In comparison with its previous iteration, the TFN, and other IFNs, the TFNA did not show an elevated risk of revision or implant failure [19]. However, a recent study reported increased complication rates with increased cutout and malpositioning of the calcar screw in comparison with the Gamma Trochanteric Nail 3 (GTN3; Stryker), which could possibly be attributable to the implant design [20].

This study suffers from several limitations. First, the study design is retrospective, which poses a risk for selection bias. It is possible that not every patient with MMPA after initial treatment at our hospital showed up again for revision, which could lead to an underestimation of the true risk. No quantification of bone mineral density was performed prospectively. However, since all fractures occurred through simple falls, osteoporosis, as an influencing factor, must be assumed in all cases presented. Furthermore, this study is purely descriptive. Therefore, since no comparative group exists, no deductions about the reasons for MMPA in patients treated with the TFNA can be made. Lastly, this study investigated only patients in which a helical blade was used as FHE. Therefore, the results are not applicable for patient in which dynamic screws are used with the TFNA.

The results of this study might be generalizable to other cohorts of patients with pertrochanteric fracture treated with the TFNA with helical blades. However, patient characteristics might influence the risk for MMPA in different patient collectives.

## Conclusion

Medial migration of the helical blade with penetration into the acetabulum is a rare, but potentially hazardous complication of femoral nailing with the TFNA system, resulting in necessity for revision surgery and total hip arthroplasty.
